# Identification of *cis-*regulatory mutations generating *de novo* edges in personalized cancer gene regulatory networks

**DOI:** 10.1186/s13073-017-0464-7

**Published:** 2017-08-30

**Authors:** Zeynep Kalender Atak, Hana Imrichova, Dmitry Svetlichnyy, Gert Hulselmans, Valerie Christiaens, Joke Reumers, Hugo Ceulemans, Stein Aerts

**Affiliations:** 1 Laboratory of Computational Biology, VIB Center for Brain & Disease Research, Leuven, Belgium; 2 Department of Human Genetics, KU Leuven, Leuven, Belgium; 3 Discovery Sciences, Janssen Research & Development, Turnhoutseweg 30, 2340 Beerse, Belgium

**Keywords:** *cis*-regulatory mutations, Whole-genome sequencing, Gene regulatory networks, Cancer genomics

## Abstract

The identification of functional non-coding mutations is a key challenge in the field of genomics. Here we introduce *μ-cis*Target to filter, annotate and prioritize *cis-*regulatory mutations based on their putative effect on the underlying “personal” gene regulatory network. We validated *μ-cis*Target by re-analyzing the *TAL1* and *LMO1* enhancer mutations in T-ALL, and the *TERT* promoter mutation in melanoma. Next, we re-sequenced the full genomes of ten cancer cell lines and used matched transcriptome data and motif discovery to identify master regulators with *de novo* binding sites that result in the up-regulation of nearby oncogenic drivers. *μ-cis*Target is available from http://mucistarget.aertslab.org.

**Electronic supplementary material:**

The online version of this article (doi:10.1186/s13073-017-0464-7) contains supplementary material, which is available to authorized users.

## Background

Oncogenic programs are characterized by aberrant gene expression profiles. A gene regulatory network underlying a cancer transcriptome can be considered as a perturbed stable network configuration, or as a cancer attractor state [1]. Gene expression changes leading from a normal cell to a malignant state are generally due to a series of acquired somatic mutations, which often affect proteins playing a key role in transcriptional regulation [2]. These can include mutations, amplifications, or translocations leading to an altered function or expression of transcription factors (e.g., MYC, TAL1, MITF, TP53), co-factors (EZH2, RB1, IDH1, MLL), or signaling molecules that lead to downstream alterations in transcription factor activity (e.g., RAS/RAC/RAF, KIT, PTEN, CDKN2A). More subtle changes can also occur in gene regulatory networks, which may cause fine-tuning of the emerging transcriptome rather than necessarily yielding a different attractor state. Such local network changes can involve the addition or removal of an edge in the network, affecting a single interaction between a transcription factor and a target gene. Edge perturbations can be caused by a mutation of a transcription factor binding site in a promoter or enhancer, leading to a *de novo* gain or loss of the binding site and a consequential expression change of a nearby target gene. Several examples of such perturbations are known to be associated with oncogenic programs, such as the gain of an ETS family binding site in the *TERT* promoter, the gain of a MYB binding site in a 7.5-kb upstream *TAL1* enhancer [3–5], and the recently identified gain of a MYB binding site 4 kb upstream of the *LMO1* oncogene [6]. Note that whereas these three examples occur recurrently across melanoma or liver cancer (for *TERT*) or across T-cell acute lymphoblastic leukemia (for *TAL1* and *LMO1*), they represent exceptional cases, since whole-genome sequencing, even across large cohorts such as 560 breast cancer genomes [7], failed to identify additional binding site changes that are significantly recurrent [8] (recently reviewed in [9–11]). This suggests either that *cis*-acting mutations are usually passenger mutations rather than driver mutations, or that they can occur as drivers at diverse positions, spread across hundreds of kilobases affecting the regulation of a target gene. The latter would render current cohort sizes underpowered and would require different approaches to identify causal *cis*-regulatory mutations and their downstream consequences.

Computational predictions of a gain or loss of a transcription factor binding site can be performed by scoring the reference and mutated sequence with a position weight matrix (PWM) of the candidate factor [12, 13]. This results in a “delta” PWM score, to which an arbitrary threshold can be applied to decide whether the gain or loss is strong enough. Such an approach is implemented in various bioinformatics tools, such as FunSeq2 [14] and OncoCis [15]. However, as position weight matrices are notorious for producing false positive predictions, the delta score also results in an excess of false positive gains or losses of binding sites. A possible solution to this problem is to take the context of the binding site into account, i.e., the encompassing regulatory region (promoter or enhancer). For example, the gain of a MYB binding site in a random genomic position may not lead to *de novo* enhancer activity, whereas such a gain in the context of RUNX binding sites (MYB and RUNX bind together to leukemia enhancers) may cause ectopic enhancer activity [16]. Computationally, this solution depends on training more complex enhancer models, for example, based on k-mer support vector machines [17], random forests [16], or deep learning (deepSEA [18]). The main limitation of this approach is the dependence on high-quality training data to construct accurate enhancer models [16].

Here we investigate how “personalised” gene regulatory network reconstruction can be used to identify specific candidate *cis*-regulatory driver mutations in cancer genomes. Gene regulatory network inference is a common technique that has provided insight into master regulators in many cancer types [19–21] and the targets they regulate. Here, we exploit gene regulatory networks for the prioritization of non-coding mutations. Particularly, by first identifying the master regulators operating in a cancer sample, we can identify those non-coding mutations that generate *de novo* targets of these master regulators. We have developed an online tool to streamline this process, called *μ-cis*Target, and we demonstrate the use of *μ-cis*Target on known cases of *TERT* promoter and *TAL1* enhancer mutations. Finally, we predict new *cis*-regulatory mutations in ten cancer cell lines for which we sequenced the genome, transcriptome, and epigenome.

## Methods

### Analysis of melanoma whole genomes for *TERT* promoter mutations

Expression data (Z-scores across all sequenced cancers in The Cancer Genome Atlas (TCGA)) for seven melanoma samples (Additional file 1: Table S1) with *TERT* promoter mutations based on [22] were downloaded from Cosmic (v74). The signatures per sample (i.e., genes that have an expression Z-score above 1) were analyzed by iRegulon to build the personalized gene regulatory networks (using the following parameters: motif collection 19 K, putative regulatory region centered around TSS [20 kb, 10 kb, 500 bp], motif rankings database across 10 and 7 species, NES threshold = 3, ROC = 0.03, rank threshold = 5000).

Raw sequence data (bam files) were available for six out of seven melanoma samples via the GDC Data Portal (Legacy Archive). We performed somatic mutation calling with VarScan [23] (command *somatic* with minimum variant allele frequency of 0.1, and a minimum coverage of five and two reads in tumor and normal samples, respectively). Non-coding mutations associated with melanoma-related or cancer driver genes were scored with MotifLocator for master transcription factors obtained in the previous step. The list of candidate *cis*-GoF mutations was filtered further using topologically associating domains (TADs) from 21 human cell lines and tissues [24].

### Whole-genome-sequencing on ten cell lines

A549, COLO-205, and PC-3 cell lines were sequenced with Complete Genomics (CG), DU-145, OVCAR-3, SKOV-3 were sequenced with Illumina, and HCT-116, HT-29, MDA-MB-231, and SK-MEL-5 were sequenced with both technologies. CG sequencing was performed by the service provider using a proprietary sequencing-by-ligation technology. CG also performed primary data analysis, including image analysis, base calling, alignment, and variant calling. CG variants were further filtered with a depth of coverage threshold of 10, mutation coverage threshold of 5, and variant allele frequency threshold of 0.20. Illumina sequencing was done in accordance with the manufacturer’s protocol. Primary data analysis was performed with the manufacturer’s software Casava (v1.8.2). Illumina variants were further filtered with a depth of coverage threshold of 10 and mutation coverage threshold of 5. Variant calls were intersected for samples that were sequenced with both technologies. Coding variants were subtracted using protein coding exon locations from GENCODE (v19). Variants were annotated as non-SNPs or SNPs using dbSNP build 144 [25].

### Cell line-specific regulatory data

H3K27ac ChIP-seq data for six of ten cell lines were obtained from the Gene Expression Omnibus (GEO) and literature: A549 and HCT-116 (GSE31755) [26], HT-29 (GSE53602), PC-3 [27], MDA-MB-231 [28], SK-MEL-5 (GSE60666) [29]. For the remaining four cell lines (OVCAR-3, SKOV-3, DU-145, Colo-205), ChIP-seq was performed in this study. These cell lines were a kind gift from the following laboratories: OVCAR-3 and SKOV-3 from the laboratory of Gynaecological Oncology, KU Leuven (head, Frédéric Amant); COLO-205 from the laboratory of Molecular Digestive Oncology, KU Leuven (head, Sabine Tejpar); and DU-145 from the laboratory of Lipid Metabolism and Cancer, KU Leuven (head, Johan Swinnen). The cell lines were grown to ~ 85% confluence per 15-cm dish. A total of 20 million cells per sample were collected, yielding ~ 20 fractions of chromatin. ChIP samples were prepared following the Magna ChIP-Seq preparation kit using at least two chromatin fractions and 2–2.5 μg of antibody per fraction. Anti-histone H3 acetyl K27 antibody (ab4729, Abcam) was used for ChIP. Per sample, 5–30 ng of precipitated DNA or input was used to perform library preparation according to the Illumina TruSeq DNA Sample preparation guide. In brief, the immunoprecipitated DNA was end-repaired, A-tailed, and ligated to diluted sequencing adapters (1/100). After PCR amplification (15–18 cycles) and bead purification (Agencourt AmpureXp, Analis), the libraries with fragment size of 300–500 bp were sequenced using the HiSeq 2500 (Illumina). Sequence reads were mapped to the reference genome (hg19-Gencode v18) using Bowtie2 2.1.0 and narrow peaks were called using MACS2 algorithm (q-value < 0.001) [30]. Then the peaks less than 350 bp from each other were merged. Additionally, we used peak calls from the ChIP-Atlas database for 433 regulatory datasets (transcription factor and chromatin ChIP-seq) across seven cell lines (Additional file 1: Table S2; http://chip-atlas.org).

### Selection of cancer type-specific transcription factors and target genes

Lists of cancer type-specific genes were extracted from NCBI Gene database (http://www.ncbi.nlm.nih.gov/gene; Additional file 1: Table S3). Next, a collection of 1050 known cancer driver genes compiled from different resources [31–36] was used to further annotate the cancer type-related genes (Additional file 1: Table S3).

### Cell line-specific gene signatures and identification of sample-specific master regulators

Gene expression data (Z-scores) per cell line were obtained from the Cosmic Cell Lines project (http://cancer.sanger.ac.uk/cell_lines). Cell line-specific gene sets were created by selecting genes that have an expression Z-score above 1. The signatures were analyzed by iRegulon to build the personalized gene regulatory networks (using the following parameters: motif collection 19 K, putative regulatory region centered around TSS [20 kb, 10 kb, 500 bp], motif rankings database across 10 and 7 species, NES threshold = 3, ROC = 0.03, rank threshold = 5000). Transcription factors that are cancer type related and expressed in the cell line (Z-score above 1) were considered as master regulators of the corresponding cell line (Table 1).

**Table 1:** Sample-specific master regulators predicted from sample-specific gene signatures

Cell line	Cancer type	Number of master TFs/motifs	Master TFs^a^	Number of cancer-type relevant or driver genes associated with master TFs
HCT-116	Colon	3/42	**ATF3**, DDIT3, NFE2L2	347
HT-29	Colon	15/254	**ETS2**, FGF19, GPD1, HDAC1, HNF1A, **KLF4**, **KLF5**, KLF6, MZF1, NR1H3, PPARG, **RELA**, **RXRA**, **SP1**, ZBTB7A	448
MDA-MB-231	Breast	4/111	**ETS1**, ETV1, **FOSL1**, **STAT5A**	419
SK-MEL-5	Melanoma	20/211	**BAX**, CAT, CTCFL, CTNNB1, E2F3, **ETV4**, **HHAT**, MITF, MXI1, **NR4A1**, **OLIG2**, PAX3, PIR, PPARGC1A, RAB7A, **RUNX3**, **SNAI2**, **SOX10**, TBX2, TFAP2A	263
DU-145	Prostate	4/99	**FOSL1**, **HOXA1**, SETDB1, **YY1**	345
SKOV-3	Ovarian	1/31	**ZEB1**	284
OVCAR-3	Ovarian	2/62	**EGR1**, **FOS**	255
A549	Lung	3/67	**CEBPB**, NFE2L2, TP63	304
COLO-205	Colon	7/160	BCLAF1, **CLOCK**, ELF3, **HNF1A**, **HOXB13**, **PITX1**, **TFF3**	445
PC-3	Prostate	7/216	ELK1, ETV1, **ETV4**, **FOSL1**, **HOXB13**, MTHFD1, SPDEF	427

The table lists the number of predicted master regulators per cell line (together with the number of motifs directly associated with these transcription factors (TFs); the names of the master TFs (bold indicating gains), and finally the number of candidate over-expressed cancer type-related or driver genes (near these genes we score mutations for motif gains)

### Detection of candidate mutations

The non-coding mutations were assigned to genes using the GREAT tool (up to 1 Mb) [37]. Only the non-coding mutations associated with genes that are a) over-expressed in the cell line (expression Z-score above 1) and b) relevant to the cancer type or on the list of known cancer drivers were scored by MotifLocator [13].

All the motifs corresponding to master transcription factors from the personalized gene regulatory networks were tested to reveal if any mutation from the matched cell line caused a gain of any of these motifs.

### Annotation of mutations using TADs

The assignment of the mutations to the potential target genes within 1 Mb is further annotated using a large dataset of TAD boundaries generated for 21 samples (14 human tissues and seven human cell lines) [24]. In the output file, information on whether the mutation and the gene fall between the boundaries of a certain sample is provided, i.e., if the mutation is in the same TAD region as the promoter of the gene. This annotation can be used to further filter mutation–gene associations.

### Genome-wide screening of DNA sequences by MotifLocator

A collection of 8053 unique PWMs directly annotated to 1628 TFs was used for scoring with MotifLocator [13]. Scoring was performed at the mutation sites with a window size between 20 and 60 bp depending on the motif size (20 bp if the motif size is 15 bp or less, 30 bp if the motif size is between 16 and 25 bp, and 60 bp if the motif size is larger than 25 bp). The variants with mutant MotifLocator score ≥ 0.90 and delta ≥ 0.1 were selected (where delta represents the difference between MotifLocator scores of mutant and wild-type sequences).

### Validation of candidate mutations using matched transcriptome and epigenome data

RNA-seq data (bam files) were downloaded for eight (A549, COLO-205, DU-145, HCT-116, HT-29, MDA-MB-231, PC-3, SK-MEL-5) cell lines from the GDC Data Portal (Legacy Archive). Heterozygous SNPs per cell line were obtained from whole genome mutation calls by requiring at least five reads for the reference and variant allele, as well as a variant allele frequency of at least 0.10. Heterozygous SNPs were intersected for samples that were sequenced with both Illumina and CG. Allele-specific expression per cell line was calculated using MBASED one-sample analysis [38]. Any gene with an adjusted *p* value ≤ 0.05 and estimated mean allele frequency ≥ 0.6 was annotated as exhibiting allele-specific expression.

## Results

### A small number of non-coding mutations generate *de novo* oncogenic edges in driver gene regulatory networks

**Fig. 1:**
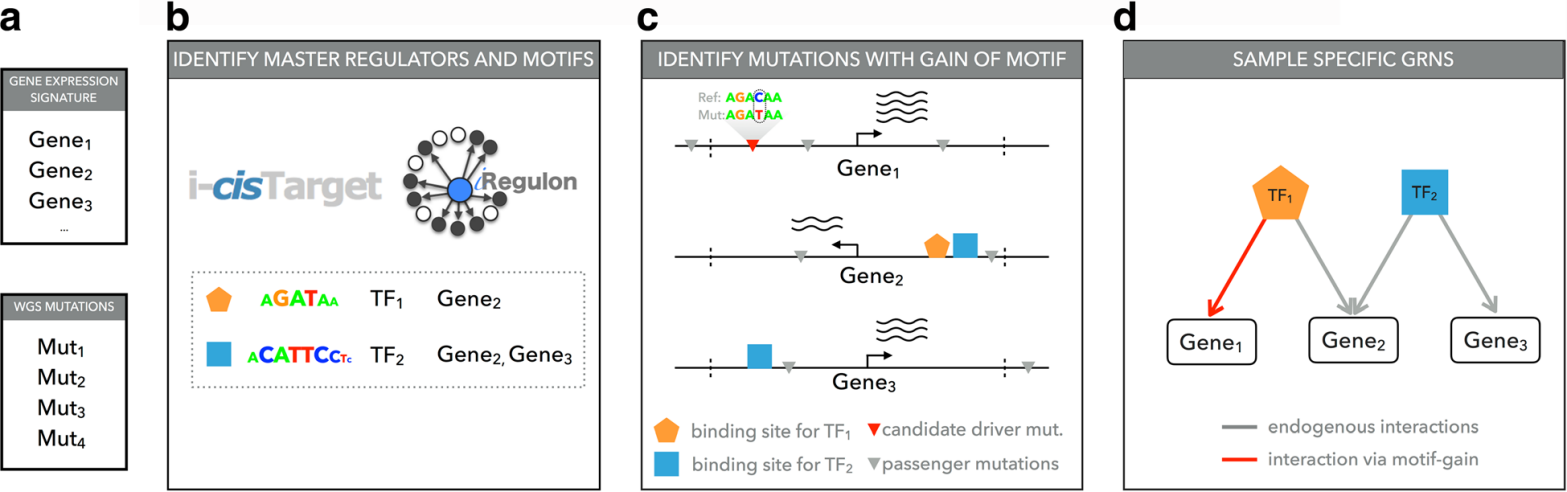
Overview of the *μ-cis*Target pipeline to predict *cis*-regulatory mutations. **a** As input *μ-cis*Target takes a gene signature and a list of genomic variations. The gene signature can be derived from the matched transcriptome of the same cancer sample, or can be a general gene signature of the matching cancer subtype. **b** Motif discovery on the gene signature yields enriched motifs and candidate transcription factors. Motif discovery can be performed using i-*cis*Target or iRegulon. **c** Variations are selected by their proximity (<1 Mb) from the genes in the input gene signature, and are scored with the motifs found under **b. d** Genes with gains of motifs for cancer type-related factors that are expressed in the sample are added to the inferred gene regulatory network (red edge). An optional filtering step selects only overexpressed cancer-related driver genes as targets (*GRN* gene regulatory network, *TF* transcription factor, *WGS* whole genome sequencing)

We developed a new computational pipeline, called *μ-cis*Target, to identify *cis*-regulatory mutations in a cancer sample, when both the whole genome sequence and the gene expression profile of that sample are available. The concept behind *μ-cis*Target is to simultaneously identify “personalized” candidate master regulators for a given cancer sample, based on the gene expression profile of the sample (and optionally combined with a ‘general’ cancer gene signature of the same cancer subtype), and to prioritize SNVs and INDELs in the non-coding genome of the sample by their likelihood to generate *de novo* binding sites for any of these master regulators (Fig. 1). Among the list of candidates, we further determine a final set of mutations by applying two filters, namely: (i) the transcription factor for which a binding site is generated is itself expressed in the sample and is related to the cancer type; and (ii) the mutation is located close (up to 1 Mb) to a target gene that is over-expressed, and within the same topologically associating domain (TAD) as the over-expressed target, it is related to the cancer type under study, and/or it is a potential driver gene (Fig. 1; see “Methods”). These criteria are largely inspired by previously published *cis*-regulatory driver mutations, such as those driving *TERT*, *TAL1*, and *LMO1* [3–6]: these oncogenes are overexpressed, and the generated binding sites are bound by (over-)expressed and cancer type-relevant transcription factors, namely GABPA for *TERT* and MYB for both *TAL1* and *LMO1*.

**Fig. 2:**
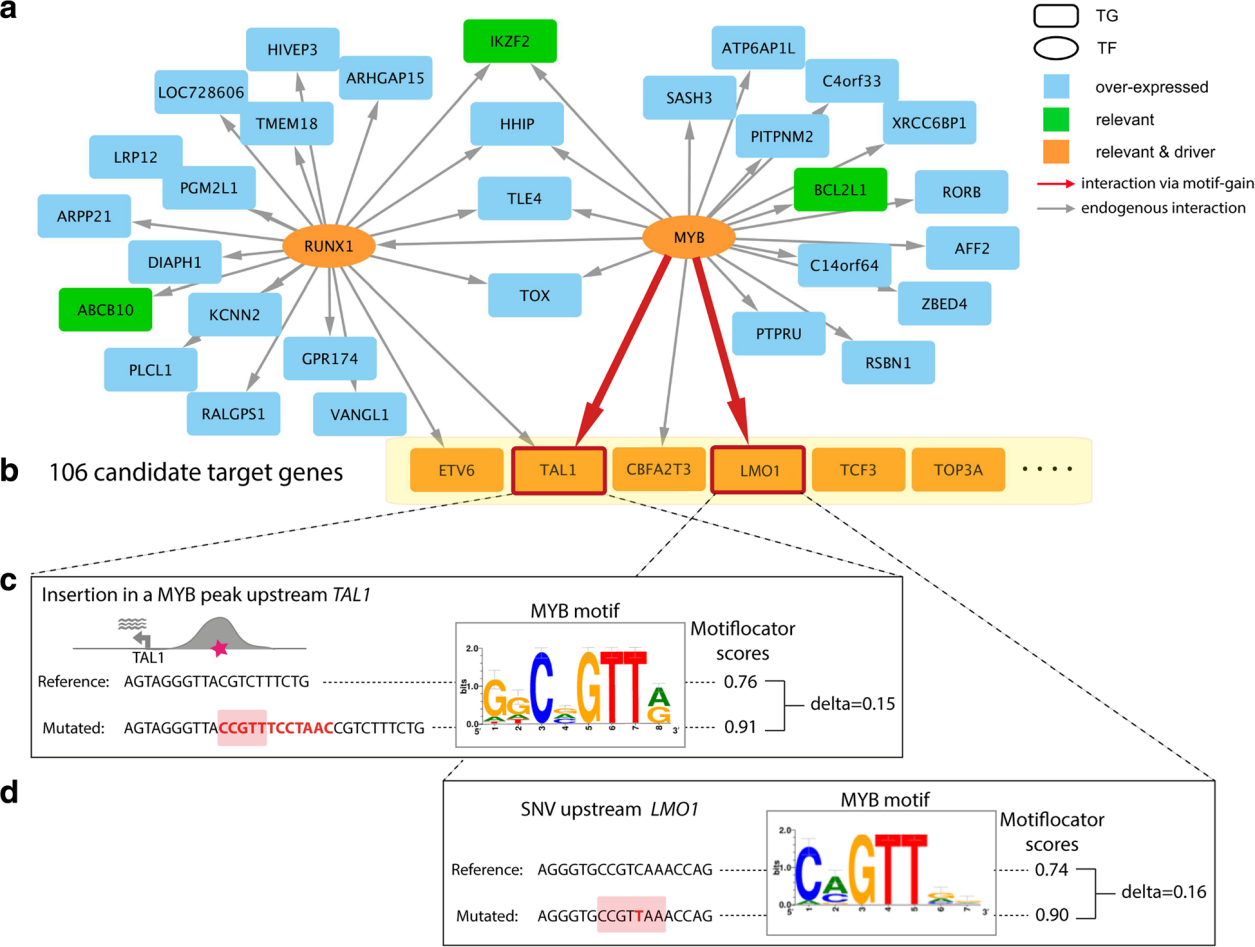
Detection of *TAL1* insertion and *LMO1* mutation in JURKAT cell line. **a** Gene regulatory network inferred from the top 500 MYB ChIP-seq peaks for the JURKAT cell line (by i-*cis*Target [65]). The top enriched motifs are directly annotated for RUNX1 and MYB transcription factors (*TF*), which are also expressed in the JURKAT cell line. Only over-expressed target genes (*TG*) in JURKAT are shown (blue nodes), of which some are moreover relevant to the leukemia cancer type (green nodes) and some are known as cancer drivers (orange nodes). The grey edges represent the link between the TF and TG based on the presence of the TF motif in a MYB ChIP-seq peak near (<1 Mb) the target gene. **b** Non-coding mutations close to candidate target genes that are overexpressed, relevant, and drivers are tested by MotifLocator to find candidate mutations that yield a motif gain. We simulated a dataset with the JURKAT insertion upstream of *TAL1* together with 67 control mutations from ten sequenced cancer cell lines (Table 1) that are found in the *TAL1* locus. Out of all 68 mutations, only the JURKAT insertion showed gain of the MYB motif, which caused a new link between *TAL1* and MYB (red arrow). **c**, **d** Details of the JURKAT insertion 7.5 kb upstream of the *TAL1* oncogene (**c**) and the JURKAT SNV 4 kb upstream of the *LMO1* oncogene (**d**), where the reference and mutated sequences are shown (the insertion/SNV is in red, the core of the motif is highlighted) together with their scores given by MotifLocator for the master MYB motifs

To illustrate how *μ-cis*Target works we first apply it to a simulated set of 67 variants spread around the *TAL1* gene (up to 1 Mb upstream or downstream), where we inserted the true driver variant that generates a *de novo* MYB binding site. In the first step of the method, we used as input the top 500 MYB ChIP-seq peaks obtained in the same sample where the variant occurs (the JURKAT cell line), which finds the MYB and RUNX1 motifs as enriched (Additional file 2: Figure S1). This analysis thus infers a candidate network with MYB and RUNX1 as master regulators (Fig. 2a). Among the 68 variants, only one generates a new binding site for any of the enriched motifs, which is the true driver mutation, with a strong gain for the MYB motif (Fig. 2b, c). We then used the same master regulators to interrogate the recently discovered *LMO1* enhancer mutation and again we could correctly predict MYB gain of motif as a result of this non-coding mutation (Fig. 2b, d). Recently, whole genome mutation calls from the JURKAT cell line became available [39], and applying *μ-cis*Target on this dataset revealed that we could correctly identify the *TAL1* enhancer mutation as a candidate *cis* gain-of-function (*cis*-GoF) mutation among 4.3 million variants (Additional file 1: Table S4) (the *LMO1* enhancer mutation is not predicted as a candidate since this mutation is not present in the mutation calls).

**Fig. 3:**
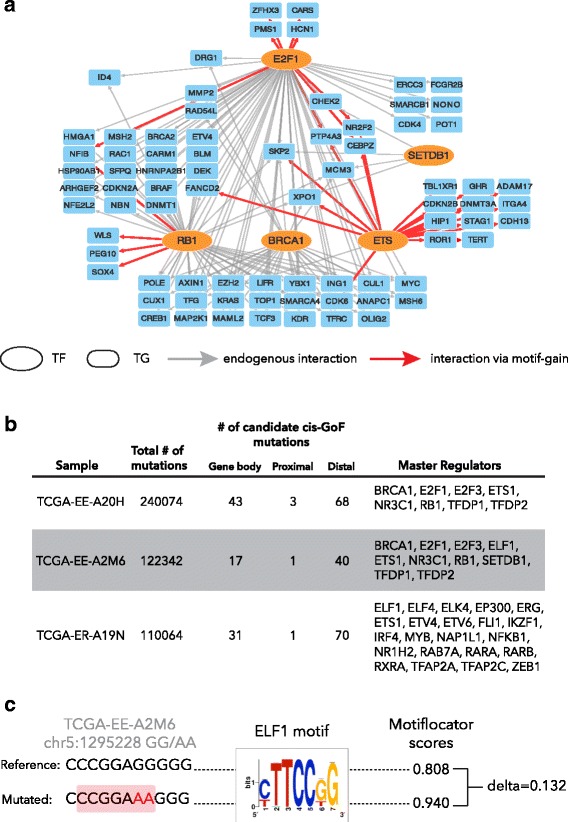
*TERT* mutation identification through a personalized gene regulatory network of TCGA-EE-A2M6. **a** Gene regulatory network inferred by iRegulon analysis from overexpressed genes (Z-score ≥ 1) of melanoma sample TCGA-EE-A2M6. Among the enriched motifs are directly annotated motifs for ETS transcription factors (*TF*; ELF1, ETS1), BRCA1, E2F family TFs (E2F1, E2F3, TFDP1, TFDP2), RB1, and SETDB1 (for simplicity the network is drawn only with cancer driver TFs; for a full list of predicted master regulators for this sample see Additional file 1: Table S1). The grey edges represent the link between TFs and target genes (*TG*) based on iRegulon analysis, while red edges indicate gain of ETS motif caused by the *TERT* promoter mutations C228T and C229T. All the represented TGs are overexpressed in TCGA-EE-A2M6, associated with melanoma, and known cancer drivers. **b**Summary table for *μ-cis*Target analysis on three melanoma whole genomes. In each case *μ-cis*Target results in a manageable number of candidate *cis*-﻿GoF mutations including the ﻿*TERT*﻿ promoter mutation.**c** Detail of the mutation at the *TERT* promoter, where the reference and mutated sequences are shown (the mutation in red, the core of the motif highlighted) together with their scores given by MotifLocator for the swissregulon__hs__ELF1_2_4.p2 motif (which is directly annotated to ELF1, ELF2, and ELF4)

Next, we tested whether *μ-cis*Target could identify the well-known *TERT* promoter mutation from a sample with a fully sequenced genome (having the *TERT* mutation) and a matched transcriptome. The *TERT* promoter mutation results in a *de novo* ETS-binding site and occurs in 55% of melanoma samples [22]. We selected seven melanoma samples from TCGA with a *TERT* promoter mutation and for which expression and whole genome mutation data are available through TCGA. We asked whether *μ-cis*Target can identify the *TERT* promoter mutation in each individual sample as a candidate *cis*-GoF mutation, starting from the matched gene expression and mutation data. The first step consists of the identification of master regulators, starting from a gene signature of sample-specific up-regulated genes. For six out of the seven samples, *μ-cis*Target predicts at least one ETS family member as master regulator (Additional file 1: Table S1). The second step of *μ-cis*Target consists of identifying mutations that result in a gain of binding site near potential oncogenic drivers per sample (i.e., overexpressed genes that are either specific for the cell type or a potential driver gene; Additional file 1: Table S3). In all those cases where an ETS factor is found as a master regulator, the *TERT* promoter mutations (both C228T and C229T) are predicted as gains of ETS binding sites (Fig. 3a–c; Additional file 1: Table S1). Next, to test the specificity of our method we re-analyzed six of these melanoma samples to obtain whole genome somatic mutation calls. This revealed that only three samples had enough coverage to detect the *TERT* promoter mutation; thus, we used these three melanoma samples to predict candidate *cis*-GoF mutations (Fig. 3b). From the initial 110 K to 240 K mutations, *μ-cis*Target identified 58 to 114 candidate *cis*-GoF mutations, including the *TERT* promoter mutations. All these candidates were either within introns or distal regulatory regions, while the *TERT* promoter mutations are among the few predictions located in a gene promoter (only TCGA-EE-A20H has two other candidate mutations that are located in a gene promoter; Additional file 1: Table S5). This demonstrates that *μ-cis*Target is able to identify a manageable number of candidate functional non-coding mutations among thousands of candidates, while providing a prediction of their function in terms of sample-specific gene regulatory networks. More importantly, our results demonstrate that *μ-cis*Target can identify a functional non-coding mutation (such as the *TERT* promoter mutation) in a sample-centric manner without requiring recurrence across a large cohort.

### Application of *μ-cis*Target to ten re-sequenced cancer cell lines

After validating *μ-cis*Target on the *TAL1*, *LMO1*, and *TERT* mutations, we analyzed ten widely used cancer cell lines as a discovery set (Additional file 1: Table S6). We essentially implemented the same strategy as in the validation cases. Namely, we first identified cell line-specific master regulators that are relevant genes per cell line using motif enrichment analysis. Next, we identified non-coding mutations in the genome of that cell line that create *de novo *binding sites for any of these master regulators, and that are near oncogenic drivers (relevant or driver genes; Additional file 1: Table S3). For the first step we obtained gene expression profiles of these cell lines from the COSMIC Cell Lines Project [40] and predicted master regulators using gene signatures of these cell lines (Table 1). For each sample, all genes expressed with Z-score above 1 (compared to all other cell lines in Cosmic Cell Line Project) are used for motif enrichment-based master regulator discovery. The initial set of predicted transcription factors was filtered for their own overexpression (Z-score above 1) and their cancer type specificity (based on Additional file 1: Table S3). Interestingly, our motif-based predictions of master regulators is supported by ChIP-seq data (when available) for 6/33 master regulators across the ten cell lines (Additional file 1: Table S6).

Transcription factors identified at this step can be linked to several signaling pathways [41] (Additional file 2: Figure S2) but two functional classes of transcription factors emerge at this step: lineage-associated transcription factors and EMT-associated transcription factors. Lineage associated transcription factors include MITF for the melanoma cell line SK-MEL-5 [42], TP63 for the lung cancer line A549 [43], KLF5 for the colon cancer line HT-29 [44], and ETS family transcription factors for prostate, colon, ovarian, and breast cancer cell lines [45–49]. This class of transcription factors is expressed at an earlier developmental stage and is reactivated during tumorigenesis. Another group of transcription factors are the EMT associated factors: FOSL1 for MDA-MB-231 and DU-145, ZEB1 for SKOV-3, FOS for OVCAR-3, and SNAI2 for SK-MEL-5. The majority of cell lines with these transcription factors as master regulators are derived from metastatic sites (SK-MEL-5, DU-145, PC-3). Of the remaining two cell lines, OVCAR-3 is derived from a chemoresistant patient [50] and MDA-MB-231 demonstrates mesenchymal cell morphology [51] and is regarded as invasive in vitro [52]. Master regulators obtained at this step also corroborate well with what is known about these cell lines. For instance, the predicted master regulators for the lung cancer cell line A549 include NFE2L2 (NRF2), which is an essential gene for cell proliferation and chemoresistance in lung cancers, and specifically in A549 since knock-down of NFE2L2 in A549 inhibits proliferation [53]. Another example involves the MDA-MB-231 cell line for which ETS factors ETS1 and ETV1, as well as FOSL1 and STAT5A/B (the motif is directly annotated for both STAT5A and STAT5B), are found as master regulators. Gene knock-down studies involving these four transcription factors in this cell line demonstrated that each of these transcription factors is essential for growth, migration, and metastatic potential of this cell line [54–57]. And lastly, it has been shown for the ovarian cancer cell line SKOV-3 that inhibition of ZEB1, which is predicted as a master regulator, hampers migration in vivo and tumor growth in vitro when xenografted in mice [58]. In conclusion, master regulator predictions seem to capture and represent oncogenic processes ongoing in these cell lines.

**Fig. 4:**
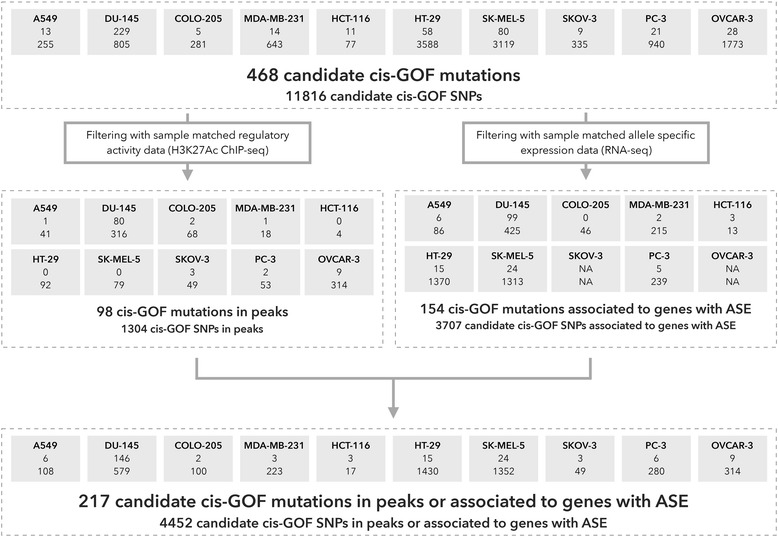
Candidate *cis*-GoF mutations in ten cancer cell lines. For each cell line the number of candidate *cis*-GoF mutations (above) and SNPs (below) are indicated within each box. Across the ten cell lines we identified 468 candidate *cis*-GoF mutations and 11,816 SNPs. Identified candidates were validated using matched regulatory (H3K27Ac ChIP-seq) and transcriptomic data, and overall 217 out of 468 candidate *cis*-GoF mutations (4452 out of 11,816 SNPs) exhibited evidence for selection. *ASE* allele-specific expression

Next, we obtained whole genome mutation calls for ten cell lines by re-sequencing them using a combination of Illumina and Complete Genomics (CG) technology (Additional file 1: Table S7). On average, the cell lines contain 1.69 million variants (SNVs and INDELS combined), of which, on average, 98% are non-coding (Table 1; Additional file 1: Table S7). For each sample, we scored non-coding mutations using sample-specific master regulators identified in the first step. Again, we defined candidate *cis*-GoF mutations as variants that generate *de novo* binding sites for any of the predicted master regulators, near oncogenic drivers. There is a high variation between the number of candidate *cis*-GoF mutations between cell lines and this correlates with the number of *somatic* coding mutations for these cell lines (r = 0.96 and *p* value < 0.05 except for HCT-116; Fig. 4; Additional file 2: Figure S3). Across all ten cell lines, *μ-cis*Target initially identifies 485 candidate mutations, and even though we assign mutations to genes in a regulatory space up to 1 Mb, almost all of them results in an association covered within a TAD (468/485). We focus on the 468 mutations associated with their targets within a known TAD affecting 290 oncogenic drivers (Fig. 4; Additional file 1: Table S8). Only 29 genes have a protein altering mutation (i.e., missense substitution, in-frame or frameshift indel) that might be associated with its overexpression and none of these genes are affected by copy number aberrations; thus, 94% of the genes are affected only by non-coding mutations (Additional file 1: Table S9). Although no genomic positions are recurrently mutated across the ten cell lines, 24 genes are recurrently affected by a *cis*-GoF mutation in two or more samples. For instance, FOXA1, which acts as a pioneering factor in prostate cancer [59], is found to be affected by *cis*-GoF mutations in the prostate cancer cell lines DU-145 and PC-3 and in the colon cancer cell line HT-29 (Additional file 1: Table S10). Moreover, two *de novo* master regulator–target gene pairs are recurrent across the ten cell lines: FOSL1–*FOXA1* in prostate cancer cell lines DU-145 and PC-3 and FOSL1–*MTRR* in the breast cancer cell line MDA-MB-231 and prostate cancer cell line DU-145. Although it may be possible that other binding site gains, located near genes that are not (yet) known as oncogenes for the cancer type under study, could play a role in the oncogenic program, we consider this as unlikely given the large amounts of cancer type-specific (and general) oncogenes that are known so far. In conclusion, *μ-cis*Target provides a short list of candidate *cis*-regulatory mutations that have a potential impact on the expression of relevant oncogenes.

### Evaluation of predicted *cis*-regulatory mutations using matched epigenomes and allele-specific expression

**Fig. 5:**
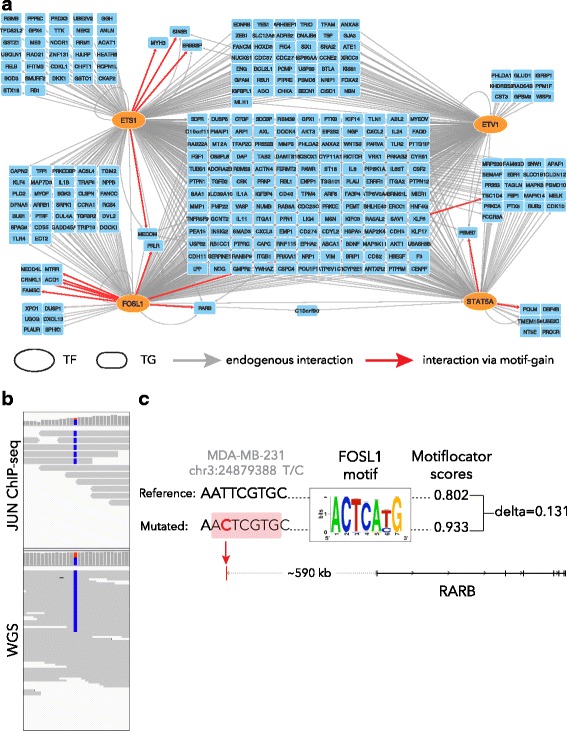
Personalized gene regulatory network of MDA-MB-231. **a** Gene regulatory network inferred by motif-enrichment analysis from overexpressed genes (Z-score ≥ 2) of breast cancer cell line MDA-MB-231. Four master regulators are identified for this cell line: ETS1, ETV1, FOSL1, and STAT5A. The grey edges represent the link between transcription factors (TFs) and target genes (TGs) based on iRegulon analysis, while red edges indicate gain of motifs caused by the *cis*-GoF. All the represented TGs are over-expressed in MDA-MB-231, are associated with breast cancer, and are known cancer drivers. **b** A *cis*-GoF mutation in a distal enhancer (590 kb upstream) of the *RARB* gene creates a *de novo* AP1 binding site (resulting in a red edge between AP1 family transcription factor FOSL1 and *RARB*). **c** IGV screenshot shows that the mutation is heterozygous in MDA-MB-231 whole genome sequence data (below) and homozygous in JUN ChIP-seq data (above)

Evaluating the potential impact of predicted oncogenic *cis*-mutations is challenging. Here, we test whether the predicted mutations may have an impact on the regulatory activity of the encompassing region. To this end, we use existing as well as newly obtained regulatory data and allele-specific expression information (as obtained from RNA-seq) for our ten cell lines. Note that previous studies have used regulatory data to filter non-coding mutations [60], but these were not sample-matched. Here we explicitly use matched regulatory data for the same sample. Overlapping candidate *cis*-GoF mutations with sample-matched H3K27Ac peaks revealed that 98 out of the 468 candidate mutations are in a potentially active regulatory region. For six out of the ten cell lines, candidate *cis*-GoF mutations are enriched in active regulatory regions (hypergeometric test *p* value ≤ 0.05; Fig. 4; Additional file 1: Table S11). The same holds true for *cis*-GoF SNPs, since for all cell lines *cis*-GoF SNPs are enriched in active regulatory regions, indicating that *μ-cis*Target can identify potentially functional variants, be it SNPs or mutations. Additionally, we queried a large set of ChIP-seq peaks against other regulatory marks and transcription factors as obtained from the ChIP-Atlas database (http://chip-atlas.org; 433 datasets for seven of our ten cell lines; Additional file 1: Table S2; see “Methods”), which revealed an additional 12 *cis*-GoF mutations that are located in a transcription factor ChIP-seq peak *in the corresponding sample* (Additional file 1: Table S8)*.* In one of these examples, a predicted gain of an AP-1 binding site is observed upstream of the *RARB* gene in the breast cancer cell line MDA-MB-231, and this site co-localizes with a JUNB ChIP-seq peak (ChIP-seq performed in MDA-MB-231). Moreover, this mutation is indeed observed in the actual reads of the JUN ChIP-seq data so it suggests that this candidate mutation creates a *de novo* AP1 binding site (Fig. 5). Nevertheless, since a few wild-type reads are detected, this site may already be bound by AP-1, and in rare cases (e.g., in HUVEC cells; Additional file 2: Figure S4) JUN is already bound to the wild-type allele.

Next, we investigated whether the predicted *cis*-regulatory mutations were present in an allele-specific manner in the expression data; in other words we checked if the mutation is associated with a gene with allele-specific expression. Using coding heterozygous SNPs from whole genome sequencing calls and RNA-seq data (which was not available for SKOV-3 and OVCAR-3), we identified genes with allele-specific expression, and this revealed that 154 of 468 candidate *cis-*GoF mutations show allelic bias in expression data (Additional file 1: Table S8). Note that our effort to identify mutations showing allelic bias in regulatory data failed since the coverage of H3K27Ac data was too low to determine variant allele frequency (80/98 candidate *cis*-GoF mutations in peaks have a depth of coverage below 5). When we expanded our search to also include SNPs, we identified 1304 SNPs in H3K27Ac peaks with a motif gain for a master transcription factor, and of these, 41 show allelic bias in the regulatory data (Additional file 1: Table S12). This illustrates that gain of important motifs can yield allele-specific regulatory activity, but very few non-dbSNP, i.e., candidate somatic mutations, were identified with this property across the ten cell lines. On the other hand, by combining regulatory activity information and RNA-seq-based allele-specific expression we found evidence of selection for 217 of 468 candidate *cis*-GoF mutations (Fig. 4). In conclusion, *μ-cis*Target can be applied to matched genome–transcriptome data, or to matched genome–epigenome data, to obtain non-coding gain-of-function mutations resulting in binding site gains for subtype-specific master regulators near overexpressed oncogenes.

## Discussion

Whole-genome re-sequencing of cancer genomes is taking a prominent place in research and the clinic. The identification and prioritization of candidate driver mutations in the non-coding genome is therefore a key challenge. Indeed, several recent studies indicate an important role for *cis*-regulatory mutations in disease, not only in cancer (e.g., *TERT*, *TAL1* [3–5, 61]), but also in complex diseases (e.g., type II diabetes FTO locus [62], Parkinson’s disease [63]) and in familial disorders (e.g., preaxial polydactyly [64]). However, other studies have highlighted that cancer *cis*-regulatory mutations are usually not recurrent across patients, except for a few exceptions such as the *TERT* promoter mutation. To reconcile these two opposing directions, we decided to step away from recurrence calculations in a cohort, but to work under the assumption that *cis*-regulatory mutations may be rarer than expected. A consequence of this assumption, if each sample harbors none or only few *cis*-regulatory mutations, is that statistical enrichment analyses may fail to provide meaningful results. Rather, the identification of functional *cis*-regulatory mutations may require a more *ad hoc* biological approach, which we explored in this study.

We are not the first to score and prioritize candidate mutations based on their putative gain (or loss) of transcription factor binding sites. In fact, most previously existing methods, such as FunSeq2, OncoCis, or RegulomeDB, use an *ad hoc* approach to annotate and filter candidate mutations based on motif loss/gain (FunSeq2, OncoCis) or on regulatory data from publicly available databases such as ENCODE (RegulomeDB). However, several important pieces of information are not utilized by previously existing tools, and are explored in our study. Firstly, the gain (or loss) of a motif is expected to be functionally relevant if the transcription factor itself is expressed in the cancer cells under study. If the transcription factor is (or was) not expressed, the gain of a binding site is not expected to be under positive selection.

Secondly, the motif gain (for an expressed transcription factor) should preferably represent a new binding site for a master regulator, meaning that this (over-expressed) transcription factor regulates other genes in the cancer cell *through this motif*. The approach we present here is, to our knowledge, the first one to take this criterion into account. Practically, to address this challenge we use a patient-specific, or subtype-specific, gene signature, in which over-represented motifs represent candidate motifs of master regulators.

Thirdly, the motif gain should either yield *de novo* regulatory activity of the encompassing enhancer or strengthen/amplify that enhancer. Such a gain of function may be visible as allele-specific bias of regulatory activity, whereby, for example, the ChIP-seq reads are homozygous for the variant.

Fourthly, the motif and enhancer gain should result in the up-regulation of a nearby oncogene so that it provides a growth advantage to the cancer cell and can be positively selected. Moreover, under this fourth piece of information, we expect that the predicted target gene is actually a known oncogene. Indeed, it is rather unlikely that previously unknown oncogenes could be discovered that are only up-regulated by a *cis*-regulatory mutation, and not by any other means (e.g., duplication, translocation, or mutation).

Taken together, our approach allows the selection of candidate mutations from whole genome sequencing data in a relatively short time since the filtering removes a large portion of the mutations (for instance, the total number of JURKAT mutations drops from 4.3 million to 200 K when we focus only on those mutations that are close to expressed and relevant genes from the predicted gene regulatory network specific for this cell line). On top of that, only motifs annotated for the transcription factors predicted as master regulators of the specific sample are used for scoring these mutations. Specifically, the time required to score one mutation and motifs directly annotated for one TF is approximately 2.5 s (e.g., 29 motifs for MYB factor); scoring 10, 100, and 1000 mutations for these motifs takes 3.6, 16, and 135 seconds, respectively.

Our approach currently focuses on gain-of-function mutations that generate new binding sites for over-expressed activators, yielding up-regulation of a nearby oncogene. Clearly, several other scenarios are not covered by our proof-of-concept analyses. These include, for example, the loss of an activator binding site near a tumor suppressor (e.g., loss of p53 binding site), the loss of a repressor binding site near an oncogene, or the gain of a repressor binding site near a tumor suppressor. We have focused in this study on the gain of an activator site near an oncogene because the currently known *cis*-regulatory driver mutations are all of this class, and this is the most conceivable and most pragmatic way to work within the context of motif discovery and gene regulatory networks. Future work is needed to address the other categories, and may reveal new types of *cis*-regulatory mutations. Currently we focus on transcription factors that are *expressed* in the sample and this may lead to problems since (i) the cut-off for expression status of a transcription factor is user-dependent (and arbitrary) and (ii) mRNA expression levels might be misleading for some transcription factors that are regulated at the protein level. This is exemplified in the *TERT* promoter case study where we identify ETS family transcription factors collectively but not the GABPA transcription factor specifically because it is only expressed (according to our cut-off) in one out of seven samples. A possible increase in the sensitivity would come from allowing motif gains for *relevant* transcription factors that are related to the cancer type even though the motif was not enriched in the input gene signature or TF﻿ wa﻿s﻿ not expressed in the sample.

Our method provides a handle on understanding non-coding mutations in the context of regulatory genomics; thus, we envisioned *μ-cis*Target not as a method for a final analysis but rather a starting point for in-depth analysis. The typical use of a strategy like we depict here can be either to annotate a cancer genome with functional information regarding *cis*-regulatory mutations or in a research context to generate a list of candidate mutations that can be further tested in a targeted screen, for example, using massively parallel enhancer-reporter assays or CRISPR-Cas9 based modulation/mutation of the candidate mutations.

## Conclusions

We present a computational framework inspired by a cancer biological viewpoint on oncogenic driver mutations. Our method can be used to identify candidate *cis*-regulatory mutations using sequence information alone, but works best on samples with combined genome and transcriptome data; while optimal results can be obtained if matching epigenome data are also available. Overall our results suggest the presence of only few *cis*-regulatory driver mutations per genome in cancer genomes that may alter the expression levels of specific oncogenes.

## Additional files

Additional file 1: Supplementary **Tables S1**–**S12**. (XLSX 427 kb)

Additional file 2: Supplementary **Figures S1**–**S4**. (PDF 1866 kb)
