# Supplementary material for: Identification of cis-regulatory mutations generating de novo edges in personalized cancer gene regulatory networks
Source: Genome Med. 2017 Aug 30;9:80. doi: 10.1186/s13073-017-0464-7 (PMC5575942; doi:10.1186/s13073-017-0464-7)
Supplement: Supplementary file 2 — Supplementary Figures S1–S4. (PDF 1866 kb) [file 13073_2017_464_MOESM2_ESM.pdf]

Supplementary Figures

Figure S1. i-*cis*Target motif enrichment results for the MYB ChIP-seq peaks on JURKAT. Distribution of the area under the curve results for all 18832 motifs tested using the top 500 MYB ChIP-seq peaks on JURKAT cell line as input set. The arrows indicate the first motif found for RUNX and MYB transcription factors together with their respective normalized enrichment scores (NES). The table shows the top ten enriched motifs with their respective origins, NES and motif logos.

Figure S2. Heatmap visualizing pathways each master regulator per cell line involved based on GeneAnalytics analysis (geneanalytics.genecards.org) [65]

Figure S3. Scatter plot showing the number of *somatic* coding mutations in 10 cell lines versus number of *cis*-GoF mutations. Here the *somatic* coding mutations were extracted from Cosmic Cancer Cell Lines project database by selecting coding mutations with "*Confirmed somatic variant*" or "*Reported in another cancer sample as somatic*" tags. The black line represents the linear fit excluding the colon cancer cell line HCT-116 and dashed grey line represents the linear fit including all cell lines.

Figure S4. IGV screenshot showing the sequence read coverage on a predicted *RARB* enhancer mutation in HUVEC FOS ChIP-seq (top and bottom tracks) together with the MDA-MB-231 JUN ChIP-seq datasets. The red arrow indicates the location of the candidate *cis*-GoF mutation in MDA-MB-231 cell line.

Figure S1

AUC distribution related to the i-cisTarget motif enrichment analysis (using a database of 18832 motifs) of the top 500 MYB ChIP-seq peaks on JURKAT cell line

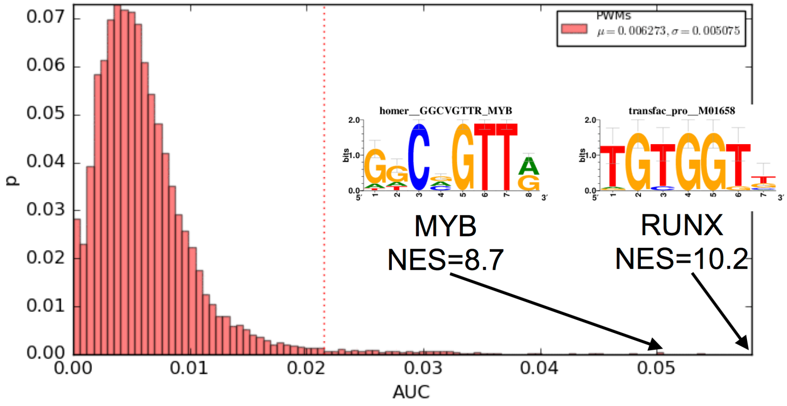

The top 10 enriched motifs

Motif	Annotated TF	NES	Logo	Cluster
transfac_pro__M01658	RUNX1	10.21668	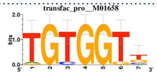	
transfac_pro__M01856	RUNX2	9.92381	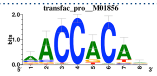	
transfac_pro__M07276	RUNX2	9.91316	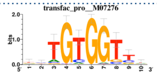	
factorbook__RUNX1	RUNX1	9.66199	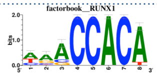	
transfac_pro__M00722	CBFB	9.40765	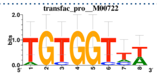	RUNX cluster
cisbp__M1837	RUNX1	9.32660	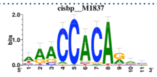	
homer__AAACCACAAA_RUNX1	RUNX1	9.32058	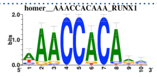	
transfac_pro__M03841	CBFB	9.30454	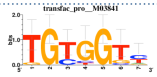	
jaspar__MA0002.2	RUNX1	9.21540	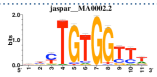	
homer__GGCVGTTR_MYB	MYB	8.70345	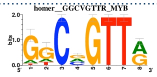	MYB cluster

Figure S2

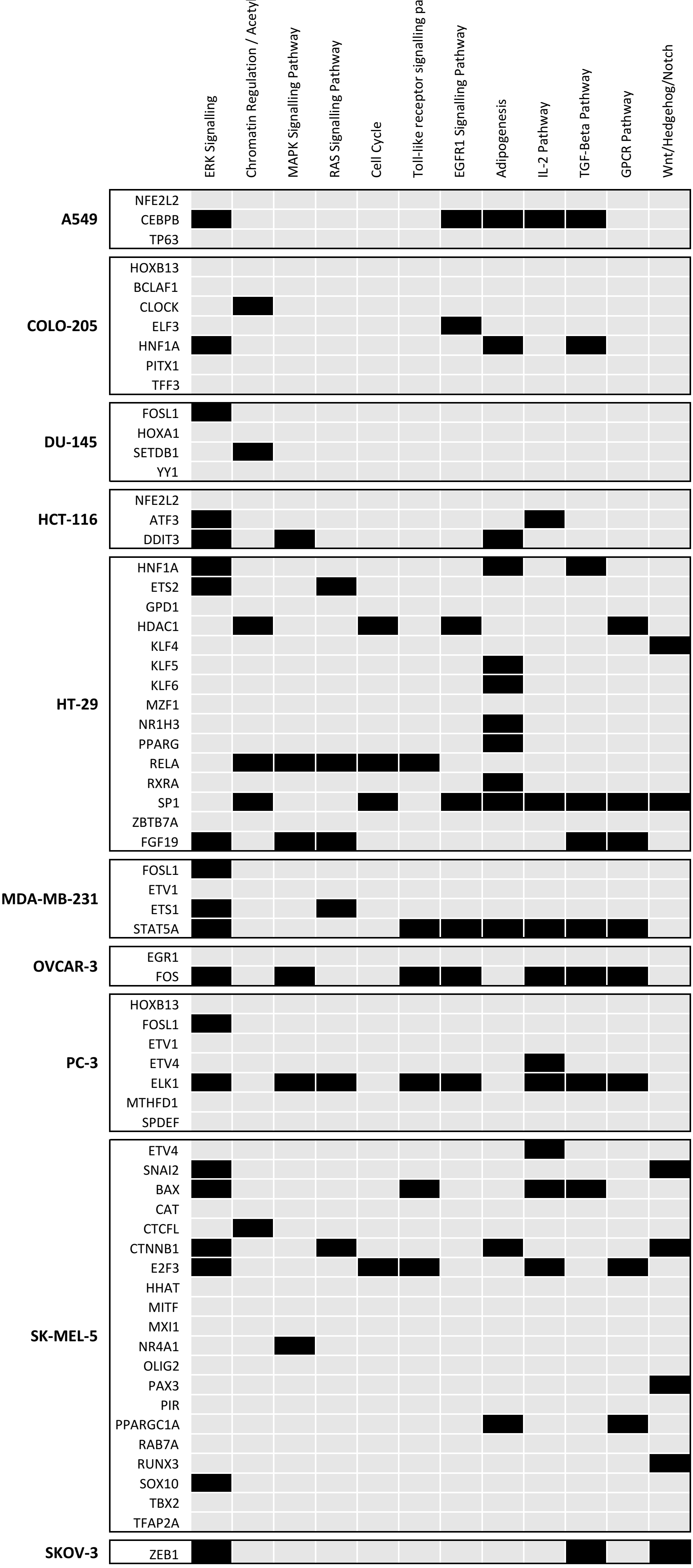
ERK Signalling
Chromatin Regulation / Acetylation
MAPK Signalling Pathway
RAS Signalling Pathway
Cell Cycle
Toll-like receptor signalling pathway
EGFR1 Signalling Pathway
Adipogenesis
IL-2 Pathway
TGF-Beta Pathway
GPCR Pathway
Wnt/Hedgehog/Notch
A549
NFE2L2
CEBPB
TP63
COLO-205
HOXB13
BCLAF1
CLOCK
ELF3
HNF1A
PITX1
TFF3
DU-145
FOSL1
HOXA1
SETDB1
YY1
HCT-116
NFE2L2
ATF3
DDIT3
HT-29
HNF1A
ETS2
GPD1
HDAC1
KLF4
KLF5
KLF6
MZF1
NR1H3
PPARG
RELA
RXRA
SP1
ZBTB7A
FGF19
MDA-MB-231
FOSL1
ETV1
ETS1
STAT5A
OVCAR-3
EGR1
FOS
PC-3
HOXB13
FOSL1
ETV1
ETV4
ELK1
MTHFD1
SPDEF
SK-MEL-5
ETV4
SNAI2
BAX
CAT
CTCFL
CTNNB1
E2F3
HHAT
MITF
MXI1
NR4A1
OLIG2
PAX3
PIR
PPARGC1A
RAB7A
RUNX3
SOX10
TBX2
TFAP2A
SKOV-3
ZEB1

Figure S3

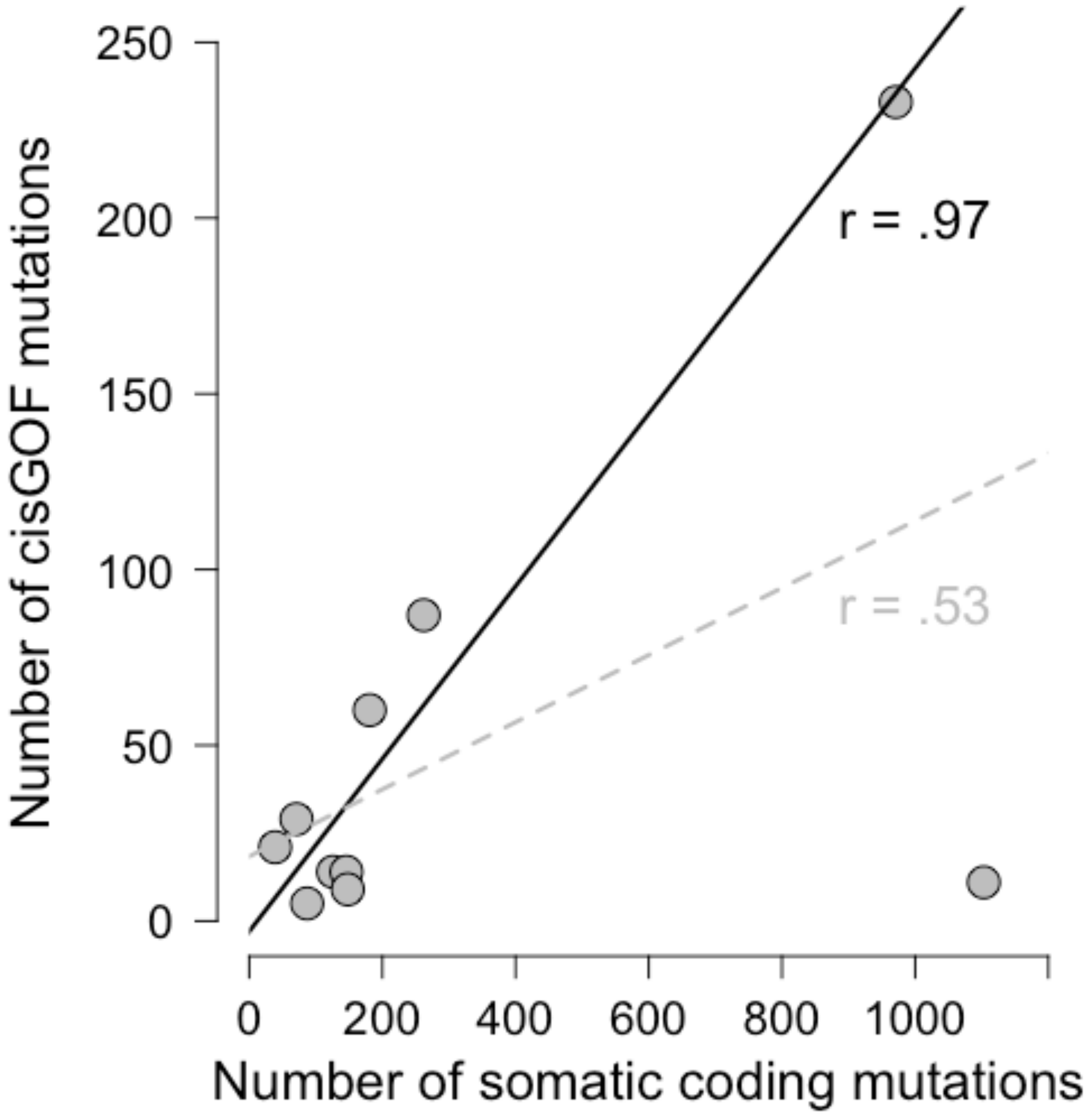

Number of cisGOF mutations
250
200
150
100
50
0
r = .97
r = .53
0
200
400
600
800
1000
Number of somatic coding mutations

Figure S4

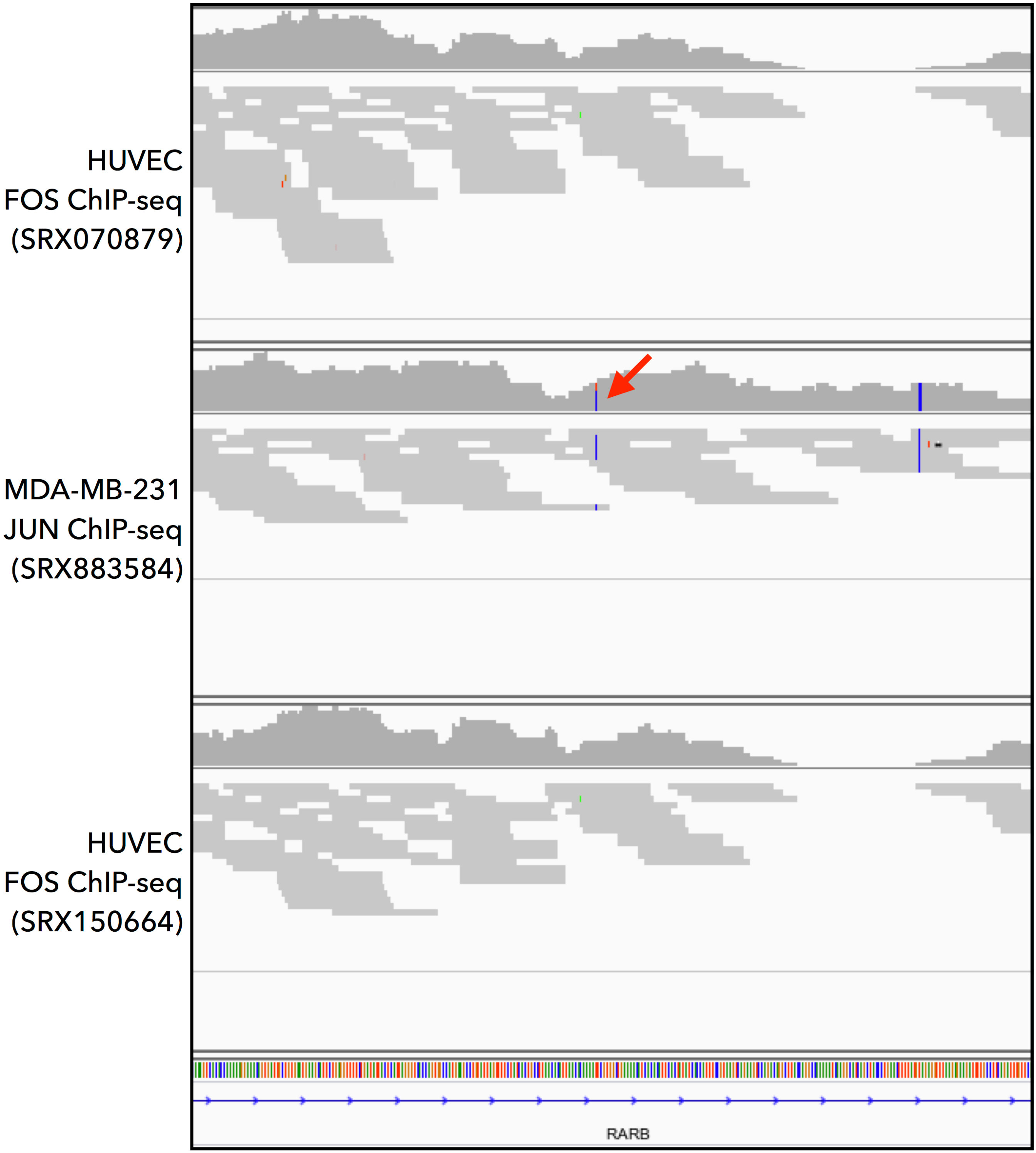
HUVEC
FOS ChIP-seq
(SRX070879)
MDA-MB-231
JUN ChIP-seq
(SRX883584)
HUVEC
FOS ChIP-seq
(SRX150664)
RARB
